# ID 97 - Brazilian HTA Database: e Laboração de Base Bibliográfica de Estudos Brasileiros na Área de ATS

**DOI:** 10.5327/2237-9622.2025.v34s1.97

**Published:** 2025-11-25

**Authors:** Gilson Pires Dorneles, Nayê Balzan Schneider, Felipe Borges Migliavaca, Bruna Marmett Jaqueline, Angela Casaes e Silva, Celina Borges Migliavaca, Maicon Falavigna

**Keywords:** Avaliação de Tecnologias em Saúde (ATS), base bibliográfica, publicações científicas, fontes de informação

## Abstract

**Introdução:**

As bases de dados bibliográficas são essenciais para obter informações de forma rápida e padronizada. Atualmente, a busca por informações brasileiras relacionadas à Avaliação e Tecnologias de Saúde (ATS) é desafiadora, e geralmente é realizada pelo acesso individual das publicações em periódicos on-line ou sites institucionais. Esta fragmentação das informações dificulta a obtenção de evidências locais, aumentando o tempo necessário e a complexidade para localizar informações, além de impactar negativamente na reprodutibilidade de buscas. Durante a última década, bases de informações de ATS têm sido elaboradas em diferentes cenários. Nesse contexto, a criação de uma base bibliográfica para estudos brasileiros na área de ATS configura-se como uma ferramenta estratégica para facilitar a disseminação e o acesso à informação. O objetivo deste trabalho foi apresentar a base bibliográfica de estudos de ATS nacionais, a Brazilian HTA Database.

**Método:**

Para o desenvolvimento da base bibliográfica, primeiramente foram identificadas revistas científicas e anais de congressos com produções brasileiras na área de ATS. Após, foi realizada a extração manual das seguintes informações de cada documento identificado: título, autores, ano de publicação, resumo, palavras-chave, link para arquivo original e DOI. O título, resumo e palavras-chave, extraídos na língua original, foram traduzidos para o inglês ou português por meio de inteligência artificial. Foram criadas variáveis adicionais para facilitar o gerenciamento dos dados: ID (identificador único da referência), fonte (de onde a referência foi extraída), tipo de documento (resumo de congresso, artigo completo, dossiê, relatório de recomendação) e referência para citação (formatada no estilo Vancouver). O desenvolvimento do sítio eletrônico foi realizado em WordPress e hospedado no domínio https://htadata.com. Atualmente, o uso da base é gratuito para fins não comerciais.

**Resultados:**

Foram identificadas como fontes prioritárias de documentos os sítios eletrônicos da ISPOR, Conitec, ANS e Rebrats, além das revistas brasileiras Jornal de Assistência Farmacêutica e Farmacoeconomia (JAFF) e Jornal Brasileiro de Economia da Saúde (JBES). A base inclui os registros destas fontes até a data de 30 de junho de 2024, incluindo 591 artigos do JAFF, 535 artigos do JBES, 871 documentos da Conitec, e 101 resumos de congresso da Rebrats; atualmente, 1.751 documentos da ISPOR e 750 da ANS estão em processo de catalogação. A busca por conteúdo pode ser conduzida de forma sistemática a partir de termos de interesse e operadores booleanos. A atualização da base, com inserção de novos documentos, será realizada mensalmente.

**Conclusão:**

A Brazilian HTA Database permite que pesquisadores, profissionais da saúde e gestores encontrem rapidamente estudos relevantes para suas necessidades. Além disso, promove a disseminação da pesquisa nacional ao permitir acesso bilíngue e centralizado da produção científica brasileira na área.

